# Non-Inferiority of a Ready-to-Drink Enteral Formula on Nutritional Status in Patients with Head and Neck Cancer: A Randomized Controlled Trial

**DOI:** 10.3390/nu18132105

**Published:** 2026-06-28

**Authors:** Manupol Tangthongkum, Ploychanok Cherngwiwatkij, Saowakon Wattanachant, Pakjira Benyapanya, Peesit Leelasawatsuk, Chaitong Churuangsuk, Kanjana Chimrung, Theepat Wongkittithaworn, Sittidet Nualnim

**Affiliations:** 1 Department of Otolaryngology Head and Neck Surgery, Faculty of Medicine, Prince of Songkla University, Hat Yai 90110, Songkhla, Thailand; ploytu911@gmail.com (P.C.);; 2 Food Science and Technology Program, Faculty of Agro-Industry, Prince of Songkla University, Hat Yai 90110, Songkhla, Thailand; saowakon.w@psu.ac.th; 3 Division of Nutrition, Songklanagarind Hospital, Faculty of Medicine, Prince of Songkla University, Hat Yai 90110, Songkhla, Thailand; 4 Clinical Nutrition and Obesity Medicine Unit, Department of Medicine, Faculty of Medicine, Prince of Songkla University, Hat Yai 90110, Songkhla, Thailand

**Keywords:** enteral nutrition, halal nutrition, head and neck cancer, nutritional support, nutritional status, randomized controlled trial, tube feeding

## Abstract

**Background/Objectives**: Malnutrition affects 20–71% of patients with head and neck cancer (HNC), owing to tumor location, treatment-induced toxicities, and systemic inflammation. Enteral nutrition is preferred when oral intake is inadequate, yet standard powdered formulas require labor-intensive reconstitution and carry a contamination risk. PSU Blen, a ready-to-drink chicken-protein-based polymeric formula, was developed to address these limitations. **Methods**: In this single-center study, thirty clinically stable patients with HNC on tube feeding were randomized 1:1 to PSU Blen or a commercial powdered polymeric formula (Blendera) for 4 weeks. The primary outcome was change in Patient-Generated Subjective Global Assessment (PG-SGA) score from baseline to week 4, analyzed under a prespecified non-inferiority framework (margin δ = 2 points, selected as a clinically acceptable upper bound based on published PG-SGA data). Secondary outcomes were handgrip strength, body weight, body mass index, serum albumin, hemoglobin, and platelet count. **Results**: The mean change in PG-SGA score was −1.13 (SD 0.92) and −0.87 (SD 1.36) in the PSU Blen and Blendera groups; the between-group difference was −0.27 points (two-sided 90% CI: −0.99 to +0.45), with the upper bound below the non-inferiority margin. Sensitivity analyses yielded consistent results. No significant between-group differences were observed for secondary outcomes, and both formulas were well tolerated, with no gastrointestinal intolerance or serious adverse events. **Conclusions**: PSU Blen demonstrated non-inferior nutritional outcomes based on the PG-SGA endpoint over 4 weeks, with comparable tolerability. Longer-term and multicenter studies are warranted to further evaluate its clinical utility and practical advantages in routine enteral nutrition practice.

## 1. Introduction

Malnutrition is highly prevalent among patients with head and neck cancer (HNC), affecting 20% to 71% of these patients [1,2,3,4]. The high burden of malnutrition is largely attributable to the tumor’s anatomical location and the adverse effects of oncologic treatment. Dysphagia, odynophagia, and taste alterations are frequent symptoms, and treatment-induced toxicities—such as mucositis, xerostomia, nausea, and vomiting—further compromise oral intake [5,6,7]. Furthermore, tumor-related systemic inflammation accelerates protein and fat catabolism, with progressive loss of muscle mass and adipose tissue [8,9]. Thus, in this population, malnutrition is associated with reduced treatment tolerance, increased infection risk, prolonged hospital stay, diminished quality of life, and worse survival outcomes [10,11,12,13]. In patients with preserved gastrointestinal function who have inadequate oral intake, enteral nutrition via nasogastric (NG) or percutaneous endoscopic gastrostomy (PEG) tubes is the preferred corrective strategy [14,15]. This approach facilitates adequate energy and nutrient intake while minimizing the risk of aspiration. In many hospitals, standard blenderized formulas remain the mainstay for tube feeding. However, these formulas pose challenges, including labor-intensive preparation, variable nutrient composition, inconsistent viscosity, and increased risk of microbial contamination, if hygiene standards are not strictly maintained [16,17,18,19]. Commercial medical liquid diets overcome some of these limitations by offering standardized nutrient content and improved microbiological safety; however, their high cost, limited variety, and—in some cases—requirement for reconstitution can reduce their practicality, particularly in resource-limited settings [20,21].

To address these limitations, PSU Blen—a ready-to-drink, nutritionally complete polymeric formula based on chicken protein—was developed. Designed to meet the Thai Recommended Daily Intakes (Thai RDI) for nutrients, PSU Blen has been approved by the Thai Food and Drug Administration and is certified as Halal, ensuring compliance with manufacturing standards and cultural acceptability. The ready-to-drink formulation of PSU Blen eliminates the need for reconstitution, reduces preparation time, and minimizes contamination risk—a concern reported in studies that used powdered and blenderized enteral formulas [16,17,18,19]—making it suitable for use in both inpatient and outpatient enteral nutrition programs. Despite these potential advantages, evidence from randomized controlled trials (RCTs) comparing PSU Blen with existing enteral formulas in patients with HNC remains limited.

This study was designed to evaluate the effectiveness of PSU Blen compared with a commercially available powdered polymeric formula, Blendera—which was selected as the active comparator because it is routinely used in clinical practice at our institution—in maintaining or improving nutritional status among clinically stable patients with HNC receiving enteral feeding. Nutritional status was assessed using the validated Patient-Generated Subjective Global Assessment (PG-SGA) tool, which is widely used in oncology. This tool integrates objective clinical findings with patient-reported nutritional symptoms into a clinically meaningful composite measure [22,23]. The secondary objectives of this study were to compare changes in handgrip strength, body weight, body mass index (BMI), and laboratory parameters (including albumin, hemoglobin level, and platelet count), as well as the incidence of feeding-related adverse events between the two groups over a 4-week intervention period. The 4-week follow-up period was selected as a pragmatic short-term interval to assess early nutritional changes while minimizing participant burden and attrition in this clinically stable outpatient population.

## 2. Materials and Methods

### 2.1. Study Design

This prospective non-inferiority randomized controlled trial was conducted at Songklanagarind Hospital, Faculty of Medicine, Prince of Songkla University, Thailand. The study protocol was approved by the Ethics Committee of the Faculty of Medicine, Prince of Songkla University (REC.66-342-13-1) and registered with the Thai Clinical Trials Registry (TCTR; TCTR20250718003). The trial was submitted to the registry during the study period; however, administrative delays during study initiation resulted in an official registration date after participant enrollment had commenced. Importantly, ethical approval was received, and the study protocol was finalized prior to the enrollment of the first participant; the trial was registered before recruitment was completed and prior to data analysis. The following key dates are reported for transparency: first participant enrollment, 1 April 2024; completion of recruitment, 9 September 2025; trial registration, 18 July 2025; and statistical analysis, 13 October 2025. The primary and secondary outcomes registered in the TCTR are fully consistent with those reported in the present manuscript, with no changes in outcomes or endpoints. The authors confirm that all ongoing and related trials for this intervention have been registered. All study procedures adhered to the ethical principles of the Declaration of Helsinki and complied with national and institutional guidelines for research involving human participants, as well as Good Clinical Practice standards.

### 2.2. Study Participants

All participants provided written informed consent prior to enrollment. Eligible participants were adult patients (≥18 years) with a confirmed diagnosis of HNC who had completed standard oncologic treatment and were in the follow-up phase at Songklanagarind Hospital. All participants were on enteral nutrition delivered via a pre-existing feeding tube—either NG or PEG. Patients were included if they were willing and able to comply with study procedures and provide accurate, reliable information. Additional eligibility criteria included an estimated glomerular filtration rate greater than 60 mL/min/1.73 m^2^ and the absence of a significant risk for refeeding syndrome, as defined by the American Society for Parenteral and Enteral Nutrition guidelines [24]. This definition included any of the following: a BMI <16 kg/m^2^; unintentional weight loss >7.5% or >10% within 3 or 6 months, respectively; negligible oral intake for seven consecutive days; oral intake of <50% of energy requirements for more than 5 days during acute illness or for more than 1 month without acute illness; clinically evident loss of subcutaneous fat or muscle mass; or the presence of high-risk comorbid conditions. Patients were eligible for study inclusion if baseline laboratory parameters for refeeding syndrome surveillance were within normal limits. Patients with a history of neuropathy or stroke that could interfere with handgrip strength measurement were excluded.

Patients were ineligible if they had a known allergy or intolerance to any ingredient in the study products. PSU Blen contains chicken, maltodextrin, green bean, rice bran oil, soy protein isolate, and salt, whereas Blendera contains maltodextrin, sugar, soy protein isolate, rice bran oil, fructooligosaccharide, sodium caseinate, and medium-chain triglyceride oil. Patients with diabetes mellitus were also excluded because Blendera contains sugar. Other exclusion criteria included gastrointestinal obstruction, gastrointestinal dysmotility, diarrhea (more than three times per day), severe liver disease (Child–Pugh class C), renal impairment (serum creatinine > 2 mg/dL), and pregnancy or breastfeeding.

### 2.3. Randomization and Intervention

Eligible patients were randomized (1:1) to receive either PSU Blen or Blendera for a total duration of 4 weeks. Randomization was performed using a computer-generated block randomization sequence with block sizes of four. Allocation was concealed in sequentially numbered, sealed, opaque envelopes prepared by a research assistant who was not involved in patient recruitment or outcome assessment. Owing to the inherent differences in product labeling, packaging, and preparation, the blinding of participants and caregivers was not feasible; however, outcome assessors remained blinded to group allocation to minimize bias. The PG-SGA tool was administered using the validated Thai-language version [25]. An independent nurse who was not a research team member was available to assist participants who had difficulty understanding individual questionnaire items, thereby minimizing potential interviewer influence on participant responses. The participants received only the assigned formula as a meal replacement, without any additional food or nutritional supplements. PSU Blen is a ready-to-drink blenderized tube-feeding product, whereas Blendera is a polymeric powdered formula that requires reconstitution with water before administration. Both formulas had an energy density of 1 kcal/mL and were administered in prescribed volumes of 300–400 mL divided into three to four daily feedings to achieve a total energy intake of 30–35 kcal/kg/day. The nutrient composition of both formulas is summarized in Table 1. Enteral feeding was administered using the bolus feeding method through the patients’ existing feeding tubes. Most participants received feeding with assistance from caregivers, although some patients could perform self-feeding. Prior to study initiation, both patients and caregivers received standardized instructions from a registered nutritionist regarding formula preparation, feeding technique, feeding schedule, and monitoring for feeding-related intolerance. Adherence to the prescribed feeding regimen was monitored using caregiver- or participant-reported diary records, which were reviewed and verified by a registered nutritionist at each scheduled study visit.

### 2.4. Data Collection

Baseline data were collected for all participants, including demographic characteristics (i.e., age, sex, weight, BMI, and underlying medical conditions), cancer type, and cancer stage according to the American Joint Committee on Cancer (AJCC) Cancer Staging Manual, 8th edition. Information regarding the type of nutritional formula received (PSU Blen or Blendera) and details of the nutritional regimen—including timing of initiation, total daily volume, and number of feedings per day—were recorded.

### 2.5. Outcome Measures

The PG-SGA was assessed at baseline and at week 4 after initiation of the nutritional intervention. Secondary anthropometric and biochemical outcomes were additionally assessed at week 2. The primary outcome was the change in continuous PG-SGA score from baseline to week 4. The PG-SGA has been validated and is available in multiple languages [25]. The PG-SGA provides a numerical score that reflects symptom burden and nutritional risk, with established triage categories: scores of 0–1 indicate no intervention required; 2–3 indicate a need for patient and family education by a dietitian, nurse, or other clinician, with consideration of pharmacologic intervention; 4–8 indicate a requirement for intervention by a dietitian in conjunction with a nurse or physician; and scores ≥9 indicate a critical need for improved symptom management and/or nutritional intervention.

Secondary outcomes included changes in handgrip strength (kg), body weight (kg), BMI (kg/m^2^), serum albumin, hemoglobin, and platelet count. Handgrip strength was measured as an indicator of muscle strength using a calibrated hand dynamometer (Takei Digital Grip Strength Dynamometer, TKK 5401, Takei Scientific Instruments Co., Ltd., Niigata, Japan). Measurements were performed in the dominant hand. The participant was seated, and the elbow was flexed at 90°; the mean of three trials was recorded. Feeding-related intolerance was assessed at each scheduled visit using the Common Terminology Criteria for Adverse Events (CTCAE), version 5.0, evaluating nausea, vomiting, bloating, and diarrhea. All gastrointestinal events were graded according to the CTCAE criteria: Grade 1, mild or asymptomatic symptoms with no intervention indicated; Grade 2, moderate symptoms requiring minimal, local, or noninvasive intervention; Grade 3, severe or medically significant symptoms requiring hospitalization or limited self-care in activities of daily living; Grade 4, life-threatening consequences requiring urgent intervention; and Grade 5, death related to the adverse event [26]. Laboratory evaluations—including serum creatinine and electrolytes (sodium, potassium, calcium, phosphorus, and magnesium)—were performed at baseline and at 2 and 4 weeks to assess safety and detect potential metabolic complications.

### 2.6. Statistical Analysis

All statistical analyses were performed using R software (version 4.5.0; R Foundation for Statistical Computing, Vienna, Austria). In accordance with the CONSORT extension for non-inferiority trials, both intention-to-treat (ITT) and per-protocol analyses were conducted and required to support the non-inferiority claim. The ITT population comprised all randomized participants analyzed according to their assigned group, whereas the per-protocol population included only participants who completed the 4-week intervention with available outcome measurements. Continuous variables are summarized as the mean ± standard deviation (SD) or median with interquartile range (IQR), as appropriate, and categorical variables are summarized as numbers and percentages (n, %). Baseline characteristics were summarized. Between-group comparisons at baseline were explored using the independent *t*-test or Wilcoxon rank-sum test for continuous variables and the chi-square or Fisher’s exact test for categorical variables, as appropriate.

The study was designed under a non-inferiority framework based on PG-SGA score assumptions. The sample size was calculated for a non-inferiority parallel-group design using the PG-SGA score. Assumptions included an SD of 1.07, derived from previously reported PG-SGA data in oncology populations (Tian et al. [27]), an anticipated true difference between groups (ε) of 1 point, and a non-inferiority margin (δ) of 2 points on the PG-SGA scale. A one-sided type I error rate of α = 0.05 and statistical power of 80% (β = 0.20) were used with a 1:1 allocation ratio, resulting in a required sample size of 15 participants per group. A 2-point margin was considered clinically interpretable because PG-SGA scores are linked to established nutrition triage thresholds (0–1: no intervention; 2–3: nutrition education; 4–8: dietitian intervention; ≥9: critical intervention) [22,23]. In addition, previous studies in head and neck cancer have suggested that relatively small differences in PG-SGA score may be associated with clinically meaningful outcomes, including treatment-related morbidity and nutritional outcomes [28]. Therefore, a 2-point margin was selected as a clinically acceptable upper bound for assessing non-inferiority in this short-term nutritional comparison.

The primary endpoint, change in PG-SGA score from baseline to week 4, was analyzed using an independent *t*-test on change scores. Because higher PG-SGA scores indicate greater nutritional risk, non-inferiority of PSU Blen relative to Blendera was concluded if the upper bound of the two-sided 90% confidence interval for the between-group difference (PSU Blen − Blendera) was below the prespecified margin of δ = 2 points. Prespecified sensitivity analyses included an analysis of covariance (ANCOVA) on the week-4 PG-SGA score adjusted for baseline and an unadjusted week-4 *t*-test. Missing PG-SGA data were not imputed because complete baseline and week-4 data were available for all 30 randomized participants; consequently, the per-protocol and ITT analyses were identical for the primary endpoint. Secondary outcomes were analyzed using linear mixed-effects models with fixed effects for the treatment group (PSU Blen vs. Blendera), time (baseline, week 2, week 4), and the group × time interaction, with a random intercept for participants to account for within-subject correlation. Models were fitted using restricted maximum likelihood estimation, and denominator degrees of freedom were approximated using the Satterthwaite method. Model assumptions were assessed using residual-versus-fitted and normal Q–Q plots. When deviations from normality were observed, outcomes were log-transformed; if assumptions remained violated, rank-based mixed-effects models were used as sensitivity analyses. Estimates of group × time interactions are reported with 95% confidence intervals (CIs) and two-sided *p*-values. Longitudinal trajectories were visualized using the ggplot2 package. This study is reported in accordance with the CONSORT 2025 statement. The CONSORT checklist is provided in Supplementary Table S1.

## 3. Results

### 3.1. Participant Enrollment and Flow

**Figure 1:**
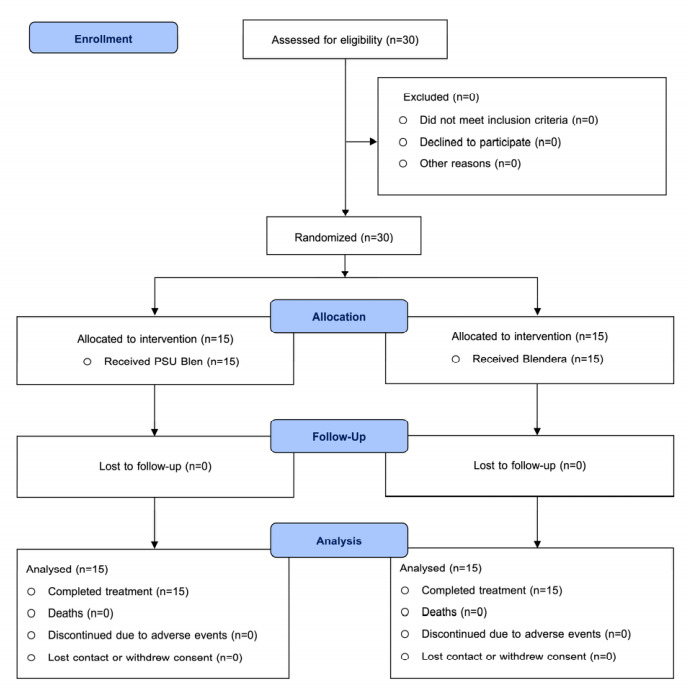
CONSORT flow diagram of patient enrollment, randomization, allocation, follow-up, and analysis.

A total of 30 eligible patients with HNC were enrolled and randomized to the PSU Blen group (n = 15) or the Blendera group (n = 15). No participants discontinued the intervention because of adverse effects during the study period. All the participants completed the 4-week intervention and had available primary endpoint data. Therefore, the ITT and per-protocol populations were identical (n = 15 per group), and both analyses yielded the same non-inferiority conclusion. The CONSORT flow diagram summarizing participant enrollment, randomization, follow-up, and analysis is shown in Figure 1.

### 3.2. Baseline Characteristics

Baseline clinicodemographic characteristics were generally comparable between the two groups (Table 2). The mean age of the study population was 58 years, and the majority were male. Hypertension and dyslipidemia were the most common comorbidities, with similar prevalence in both groups. Most participants had advanced-stage disease (Stage III or IV) according to the AJCC Cancer Staging Manual, 8th edition. However, age differed significantly between groups (PSU Blen: 53.3 years vs. Blendera: 62.7 years; *p* = 0.045), and numerical imbalances were observed in sex distribution, body weight, and BMI. These imbalances were addressed through additional ANCOVA sensitivity analyses adjusted for clinically relevant baseline variables (Table 3). The adjusted analyses yielded conclusions consistent with the primary analysis.

### 3.3. Nutritional Outcomes

At baseline, the mean PG-SGA score was 2.20 (SD 0.78) in the PSU Blen group and 2.00 (SD 0.66) in the Blendera group. At week 4, the mean change in PG-SGA score from baseline was −1.13 (SD 0.92) and −0.87 (SD 1.36), respectively. The estimated between-group difference (PSU Blen − Blendera) was −0.27 points (two-sided 90% CI: −0.99 to +0.45). Because the upper bound of the 90% CI was well below the prespecified non-inferiority margin of +2.0 points, non-inferiority of PSU Blen relative to Blendera for the PG-SGA endpoint was established. Sensitivity analyses, including ANCOVA adjusted for age, sex, body weight, and baseline PG-SGA and an unadjusted week-4 *t*-test, yielded congruent estimates, all confirming non-inferiority (Table 3; Figure S1).

**Figure 2:**
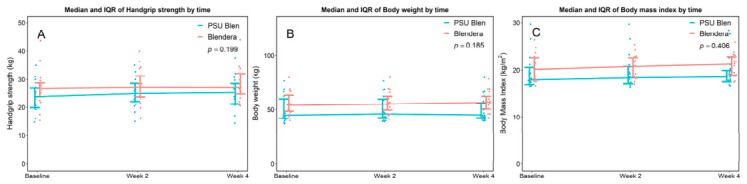
Changes in (**A**) handgrip strength, (**B**) body weight, and (**C**) body mass index (BMI) in the PSU Blen and Blendera groups at baseline, week 2, and week 4. Individual participant data points are shown alongside group medians and interquartile ranges.

**Figure 3:**
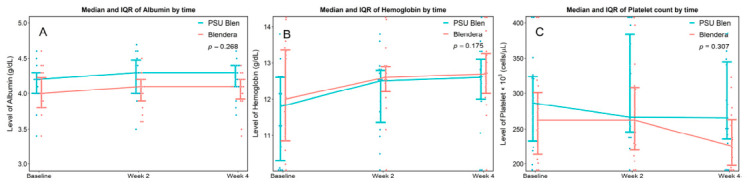
Changes in (**A**) serum albumin, (**B**) hemoglobin levels, and (**C**) platelet count in the PSU Blen and Blendera groups at baseline, week 2, and week 4. Individual participant data points are shown alongside group medians and interquartile ranges.

Longitudinal trends in anthropometric parameters—including handgrip strength, body weight, and BMI—are shown in Figure 2. Overall, both groups demonstrated similar trajectories over the 4-week follow-up period. Similarly, laboratory parameters related to nutritional status—including serum albumin, hemoglobin, and platelet count—remained generally stable during follow-up in both groups (Figure 3).

Estimated between-group differences in changes in anthropometric and laboratory values over the 4-week intervention period are summarized in Table 4. The between-group differences and corresponding 95% CIs reported in Table 4 are presented as estimates of effect magnitude for the secondary outcomes. Overall, the observed differences were modest and of limited clinical relevance. Handgrip strength increased modestly in both groups, with no significant between-group difference (−1.79 kg, 95% CI: −5.82 to 2.23; *p* = 0.382). Changes in body weight and BMI similarly showed no statistically significant between-group differences (−0.37 kg, 95% CI: −1.27 to 0.54; *p* = 0.427, and 0.02 kg/m^2^, 95% CI: −0.47 to 0.50; *p* = 0.948, respectively). Laboratory values were similar in both groups, with a modest increase in hemoglobin levels and no significant between-group differences in hemoglobin, serum albumin, or platelet count (Table 4).

### 3.4. Adverse Effects

Throughout the 4-week intervention period, no gastrointestinal symptoms, including nausea, vomiting, bloating, or diarrhea, were reported in either group. Based on the CTCAE v5.0 assessment, no gastrointestinal-related adverse event category greater than grade 1 was recorded in either group. Laboratory safety assessments, including serum creatinine and electrolyte levels (sodium, potassium, calcium, phosphorus, and magnesium), were also evaluated. Serum potassium levels were statistically higher in the Blendera group compared with the PSU Blen group at week 4; however, potassium levels in both groups remained within the normal clinical reference range. Overall, the group-level laboratory values remained within the normal reference ranges at week 4 following the initiation of nutritional support (Table 5). No serious adverse events were observed during the study period.

## 4. Discussion

In this 4-week randomized controlled trial of clinically stable patients with HNC on tube feeding, PSU Blen was non-inferior to a commercially available powdered polymeric formula (Blendera) for the change in continuous PG-SGA score from baseline to week 4. The estimated between-group difference was −0.27 points (two-sided 90% CI: −0.99 to +0.45), with the upper CI bound well below the prespecified non-inferiority margin of +2.0 points; this conclusion was supported by congruent estimates across prespecified sensitivity analyses (baseline-adjusted ANCOVA; unadjusted week-4 *t*-test). No statistically significant between-group differences were observed in secondary anthropometric outcomes (handgrip strength, body weight, BMI) or biochemical markers (serum albumin, hemoglobin, platelet count). Both formulas were equally well tolerated, with no gastrointestinal intolerance or serious adverse events reported. Although serum potassium was statistically higher in the Blendera group at week 4 than in the PSU Blen group, the values in both groups remained within the normal reference range, the potassium contents of the formulas were comparable, and no potassium-related clinical events occurred. Therefore, the clinical significance of this difference appears limited.

Our findings are consistent with those of previous studies that evaluated the effectiveness of enteral nutrition in patients with cancer and other chronic conditions. Vieira et al. [29] reported no clinically significant differences in nutritional outcomes between commercial polymeric formulas and homemade or blenderized food diets, provided that the energy requirements were met and diet administration was guided by dietitians. Chloupek and Jurkiewicz [30] and Schmidt et al. [31] found no advantage in clinical or laboratory outcomes when comparing commercial polymeric formulas with hospital-prepared liquid diets in patients with HNC or neurological conditions. Xavier de Melo et al. [32] and Tanchoco et al. [33] demonstrated that the type or category of nutritionally complete enteral formula—whether homemade enteral preparation, standard, ready-to-use, or reconstituted powdered—did not significantly influence the nutritional status of patients receiving home enteral nutrition or with stable chronic disease. However, direct comparisons across these studies are limited by differences in study populations (cancer type and treatment phase), formula types, baseline nutritional risk, and follow-up duration. The lack of observed differences in our trial may be explained by several factors. First, both PSU Blen and Blendera are nutritionally complete standard polymeric formulas that are designed to meet the same nutrient and caloric requirements, which reduces the likelihood of major outcome divergence regardless of whether the formula is powdered or ready-to-drink. Second, our study population was clinically stable, and the majority were in a follow-up phase, rather than undergoing active chemoradiation, which could have potentially decreased the risk of rapid nutritional decline. Third, as both groups received individualized caloric and nutrient prescriptions under close dietitian supervision, the intervention may have minimized the potential for nutritional deterioration and thereby reduced the likelihood of detecting between-group differences, which likely contributed to the absence of meaningful between-group differences observed in this trial.

Despite the modest between-group differences, hemoglobin levels slightly improved in both groups during the 4-week intervention period, without deterioration in other nutritional parameters. These findings suggest that the enteral feeding regimens generally maintained hematologic status during the follow-up period. Previous studies have reported that nutritional support through enteral feeding may help maintain hematologic parameters in patients with HNC undergoing treatment. Asensi et al. [34] demonstrated that hemoglobin levels increased in patients with HNC who received targeted caloric intake through enteral nutrition during treatment. Similarly, Yang et al. [35] reported improved hemoglobin parameters in patients with HNC who received enteral feeding combined with dietary counseling. The absence of clinically meaningful between-group differences further supports the non-inferiority conclusion for short-term nutritional outcomes.

From a clinical perspective, ready-to-drink formulas such as PSU Blen may offer potential practical advantages in convenience, hygiene, and preparation standardization. Such products may reduce the need for reconstitution, potentially shorten preparation time for caregivers, and may lower the risk of microbial contamination associated with improper mixing or handling [17,20,29]. In real-world practice, such characteristics may be particularly relevant in outpatient follow-up care, home-based tube feeding, and settings where consistent formula preparation, simplified administration, and caregiver support are important considerations. However, these practical characteristics—including convenience, preparation time, preparation standardization, caregiver burden, cost-effectiveness, and microbial safety—were not directly assessed in the present study. Therefore, they should be interpreted as potential advantages rather than demonstrated findings. In the present study, no gastrointestinal intolerance symptoms were observed during the intervention period, which may partly reflect the clinically stable post-treatment population and the use of standard polymeric enteral formulations with moderate energy density (1 kcal/mL), which are generally well tolerated in clinical practice. In the context of HNC—where many patients require prolonged enteral feeding—these practical features may be particularly important, especially for patients with limited caregiver support or those at increased risk of feeding-related complications. Moreover, PSU Blen has received approval from the Thai Food and Drug Administration and is certified Halal, thereby potentially helping to address an unmet need among Muslim patients requiring enteral nutrition. Religiously and culturally appropriate enteral nutrition options remain limited in clinical practice, as many widely used commercial enteral formulas are not Halal-certified. This limitation may influence adherence, acceptability, and equitable access to standardized nutritional support in relevant care settings. However, religious acceptability, patient preference, adherence related to cultural or religious factors, and cultural suitability were not formally assessed in this study and should be evaluated in future research. From a micronutrient perspective, PSU Blen and Blendera differed in the content of several key micronutrients, including calcium, iron, zinc, vitamin D, and vitamin B12, as detailed in Table 1. Notably, the calcium content of PSU Blen was substantially lower than that of Blendera. Although no clinically relevant biochemical differences were observed during the 4-week intervention, this short follow-up period may have been insufficient to detect clinically meaningful effects or determine the long-term implications of lower calcium intake and differences in other micronutrients. This issue may be particularly relevant for patients with HNC requiring prolonged or exclusive enteral feeding, as they may already be at risk of nutritional deficiencies. Therefore, long-term calcium intake should be monitored, and calcium supplementation may be warranted to ensure nutritional adequacy in this setting. Future studies with extended follow-up and comprehensive micronutrient monitoring are needed to evaluate the long-term clinical implications of these compositional differences.

This study has several strengths. The randomized controlled design minimized selection bias and enabled a direct comparison between PSU Blen and a commonly used commercial formula. In addition, the use of multiple validated and objective measures—including PG-SGA, handgrip strength, anthropometric parameters, and biochemical markers—provided a comprehensive evaluation of nutritional status. The reliability of the findings was further supported by the standardized nutritional intervention, as participants received only the assigned formula without additional food or supplements during the study period. However, several limitations should be acknowledged. Although ethical approval and protocol finalization were completed before enrollment of the first participant, the trial was registered after enrollment had begun because of administrative delays. This limitation should be considered when interpreting the methodological transparency of the trial. Muscle function was assessed using only handgrip strength, and additional functional performance or body composition measures—such as gait speed, the Short Physical Performance Battery, the Timed Up and Go test, or bioimpedance analysis—were not assessed. In addition, quality of life, patient satisfaction, caregiver burden, and cost-effectiveness were not evaluated in this study. The study’s open-label design, necessitated by differences in product packaging and preparation, may have introduced performance bias, particularly in product administration, caregiver-dependent aspects of the intervention, and patient-reported components of the PG-SGA assessment. Furthermore, the single-center design, small sample size, baseline imbalances in age and sex distribution, and relatively short 4-week follow-up period may limit generalizability and the ability to detect clinically meaningful between-group differences or longer-term nutritional changes. The 4-week follow-up period was also insufficient to evaluate longer-term nutritional status, muscle mass, micronutrient status, or clinical outcomes relevant to chronic enteral nutritional support in patients with cancer. In addition, the relatively low baseline PG-SGA scores in both groups, consistent with the clinically stable post-treatment profile of the enrolled population, may have limited the sensitivity in detecting further nutritional improvement. PSU Blen was developed by Prince of Songkla University, the authors’ institution, which holds the associated intellectual property rights. This institutional relationship has been disclosed in the Conflicts of Interest Section and should be considered when interpreting the study findings. Additionally, the exclusion of patients with diabetes mellitus, which was necessitated because of the sucrose content in Blendera, limits the applicability of these findings to patients without diabetes. Future studies should incorporate multicenter populations with extended follow-up durations, include more comprehensive functional assessments and body composition analysis, and evaluate patient-reported outcomes, including quality of life and patient satisfaction. Moreover, assessments of practical considerations—such as preparation time, contamination risk, caregiver burden, and cost-effectiveness—would further clarify the potential advantages of ready-to-use blenderized enteral formulas in clinical practice. Notably, gut microbiota composition was not assessed in the present trial. Future studies may incorporate microbiome profiling to examine whether ready-to-drink polymeric formulas, such as PSU Blen, influence host–microbiome interactions in patients with HNC.

## 5. Conclusions

PSU Blen demonstrated non-inferior short-term nutritional outcomes compared with the control formula in patients with HNC receiving enteral feeding, based on changes in PG-SGA score over 4 weeks, with consistent findings across prespecified sensitivity analyses. Both formulas were well tolerated. These findings suggest that PSU Blen may serve as a ready-to-drink enteral nutrition alternative in clinical conditions similar to those of the present study. Larger, multicenter, longer-term trials are needed to confirm its clinical value, cost-effectiveness, safety, acceptability, and practical advantages.

## Figures and Tables

**Table 1:** Nutritional composition of PSU Blen and Blendera per 100 kcal.

Nutrient	PSU Blen	Blendera
**Energy density (kcal/mL)**	1.00	1.00
**Macronutrients**		
Carbohydrate (g)	14.00	13.39
Fat (g)	3.43	3.22
Protein (g)	4.00	3.58
**Selected micronutrients**		
Sodium (mg)	117.14	76.26
Potassium (mg)	108.38	105.86
Calcium (mg)	2.75	51.86
Phosphorus (mg)	35.86	54.24
Iron (mg)	4.00	1.01
Zinc (mg)	2.73	1.47
Vitamin A (µg)	50.53	48.31
Vitamin D (µg)	1.00	0.42
Vitamin B12 (µg)	0.30	0.57
Folic acid (µg)	27.00	37.29

Estimated daily nutrient intake was calculated by multiplying each per-100-kcal value by the prescribed daily energy intake in units of 100 kcal. For example, the multiplier for 1500 kcal/day is 15.

**Table 2:** Baseline clinicodemographic characteristics of patients in the PSU Blen and Blendera groups.

Characteristic	PSU Blen (n = 15)	Blendera (n = 15)	*p*-Value
**Sex, n (%)**			0.080 ^2^
Female	6 (40)	1 (6.7)	
Male	9 (60)	14 (93.3)	
**Age, years**, mean (SD)	53.3 (12.3)	62.7 (12.1)	0.045 ^1^
**Weight, kg**, median (IQR)	45 (42.2–59.7)	53.9 (48.5–63.1)	0.081 ^3^
**Height, cm**, mean (SD)	159.9 (8.3)	164 (6.5)	0.139 ^1^
**Body Mass Index, kg/m^2^**, median (IQR)	17.9 (16.9–20.5)	20.1 (17.9–22.6)	0.191 ^3^
**Cancer subsite, n (%)**			0.907 ^2^
Oral cavity	6 (40)	6 (40)	
Oropharynx	1 (6.7)	1 (6.7)	
Hypopharynx	4 (26.7)	3 (20)	
Glottis	2 (13.3)	4 (26.7)	
Other	2 (13.3)	1 (6.7)	
**Tumor stage**			0.818 ^2^
I	0 (0.0)	1 (6.7)	
II	1 (6.7)	1 (6.7)	
III	2 (13.3)	3 (20)	
IV	12 (80.0)	10 (66.7)	
**Underlying disease, n (%)**			
Hypertension	2 (13.3)	5 (33.3)	0.390 ^2^
Dyslipidemia	2 (13.3)	3 (20)	1 ^2^

Values are presented as the mean and standard deviation (SD) or median and interquartile range (IQR), unless otherwise stated. ^1^ *t*-test. ^2^ Fisher’s exact test. ^3^ Rank-sum test.

**Table 3:** Non-inferiority of PSU Blen versus Blendera on the Patient-Generated Subjective Global Assessment (PG-SGA) score over 4 weeks.

Variable	PSU Blen (n = 15)	Blendera (n = 15)	Difference, PSU Blen − Blendera (Two-Sided 90% CI)	Non-Inferiority
**PG-SGA score**				
Baseline, mean (SD)	2.20 (0.78)	2.00 (0.66)	—	—
Week 4, mean (SD)	1.07 (0.70)	1.13 (0.83)	—	—
**Primary analysis**				
Change from baseline, mean (SD)	−1.13 (0.92)	−0.87 (1.36)	−0.27 (−0.99 to +0.45)	Met
**Sensitivity analyses**				
Sensitivity 1: ANCOVA, adjusted for PG-SGA	—	—	−0.02 (−0.51 to +0.46)	Met
Sensitivity 2: ANCOVA, adjusted for age, sex, BW, and baseline PG-SGA	—	—	+0.35 (−0.15 to +0.85)	Met
Sensitivity 3: Week-4 score, unadjusted	—	—	−0.07 (−0.55 to +0.41)	Met

PG-SGA, Patient-Generated Subjective Global Assessment; ANCOVA, analysis of covariance; BW, body weight; CI, confidence interval; SD, standard deviation. Higher PG-SGA scores indicate greater nutritional risk. Non-inferiority was concluded when the upper bound of the two-sided 90% CI for the between-group difference was below the prespecified margin (δ = +2.0 points). “Met” indicates that the upper 90% CI bound was below δ. ANCOVA sensitivity analyses were adjusted for clinically relevant baseline variables. Body weight and BMI were not included simultaneously in the same adjusted model because BMI is derived from body weight and height, and including both variables may introduce multicollinearity. The primary and sensitivity analyses included all randomized participants. Because all the participants completed the intervention and had available primary endpoint data, the ITT and per-protocol populations were identical.

**Table 4:** Changes in nutritional and laboratory parameters over 4 weeks according to treatment group.

Parameter	PSU Blen Baseline (n = 15)	PSU Blen Week 4 (n = 15)	Blendera Baseline (n = 15)	Blendera Week 4 (n = 15)	Between-Group Difference in Change ^1^ (95% CI)	*p*-Value ^2^
Handgrip strength (kg)	23.77 (19.87–27.03)	25.30 (21.15–28.60)	26.90 (23.81–28.79)	27.23 (24.73–32.10)	−1.79 (−5.82 to 2.23)	0.382
Body weight (kg)	45.00 (42.15–59.70)	45.25 (42.50–55.25)	53.90 (48.55–63.15)	56.40 (50.60–62.15)	−0.37 (−1.27 to 0.54)	0.427
Body mass index (kg/m^2^)	17.91 (16.94–20.52)	18.59 (17.51–19.93)	20.13 (17.91–22.59)	21.23 (18.78–22.81)	0.02 (−0.47 to 0.50)	0.948
Albumin (g/dL)	4.20 (4.00–4.30)	4.30 (4.10–4.40)	4.00 (3.80–4.22)	4.10 (3.92–4.20)	0.04 (−0.20 to 0.28)	0.744
Hemoglobin (g/dL)	11.80 (10.30–12.60)	12.60 (12.00–13.10)	12.00 (10.85–13.35)	12.70 (12.15–13.25)	0.39 (−0.27 to 1.06)	0.248
Platelet count (×10^3^/µL)	287 (233–324)	266 (236–345)	262 (214–301)	226 (198–262)	15 (−40 to 70)	0.591

Values are presented as the median and interquartile range or 95% confidence interval (CI), as indicated. Between-group differences in change were calculated as PSU Blen minus Blendera. Positive values indicate greater increases or smaller decreases, whereas negative values indicate smaller increases or greater decreases in the PSU Blen group relative to the Blendera group. ^1^ Between-group difference represents the estimated treatment effect at week 4 (PSU Blen vs. Blendera) based on the group × time interaction term in the linear mixed-effects model. ^2^ *p*-values correspond to the group × time interaction in linear mixed-effects models, including treatment group (PSU Blen vs. Blendera), time (baseline, week 2, week 4), and a random intercept for participants.

**Table 5:** Laboratory parameters of patients receiving PSU Blen and Blendera at 4 weeks after the initiation of the nutritional intervention.

Parameter	PSU Blen (n = 15)	Blendera (n = 15)	*p*-Value
Creatinine (mg/dL)	0.84 (0.69–0.96)	0.78 (0.73–0.86)	0.678
Sodium (mmol/L)	138.90 (136.98–139.30)	138.30 (137.30–140.70)	0.865
Potassium (mmol/L)	3.96 (3.69–4.04)	4.31 (3.96–4.50)	0.012 ^1^
Magnesium (mg/L)	20.40 (19.62–21.78)	20.85 (19.92–22.08)	0.408
Phosphorus (mg/dL)	3.35 (3.10–3.48)	3.35 (3.00–3.65)	0.890
Calcium (mg/dL)	9.50 (9.12–9.60)	9.40 (9.17–9.78)	0.890

Values are presented as the median and interquartile range. *p*-values calculated using the Wilcoxon rank-sum test. ^1^ Statistically significant at *p* < 0.05. Normal reference ranges for adults: creatinine, 0.6–1.2 mg/dL; sodium, 136–145 mmol/L; potassium, 3.5–4.6 mmol/L; magnesium, 16–26 mg/L; phosphorus, 2.3–4.3 mg/dL; and calcium, 8.4–10.2 mg/dL.

## Data Availability

The data presented in this study are available on request from the corresponding author. The data are not publicly available due to ethical and privacy restrictions imposed by the Ethics Committee of the Faculty of Medicine, Prince of Songkla University, to protect participant confidentiality. Qualified researchers may apply for data access through the Research Ethics Committee (medpsu.ec@gmail.com).

## Supplementary Materials

The following supporting information can be downloaded at: https://www.mdpi.com/article/10.3390/nu18132105/s1; Figure S1: Forest plot of non-inferiority analyses on continuous PG-SGA score; Table S1: CONSORT checklist for randomized controlled trials [36].

## Abbreviations

The following abbreviations are used in this manuscript:

ANCOVA	Analysis of covariance
BMI	Body mass index
CI	Confidence interval
CTCAE	Common Terminology Criteria for Adverse Events
HNC	Head and neck cancer
IQR	Interquartile range
ITT	Intention-to-treat
PEG	Percutaneous endoscopic gastrostomy
PG-SGA	Patient-Generated Subjective Global Assessment
NG	Nasogastric
RDI	Recommended daily intake
RCTs	Randomized controlled trials
SD	Standard deviation
