# Supplementary material for: Non-Inferiority of a Ready-to-Drink Enteral Formula on Nutritional Status in Patients with Head and Neck Cancer: A Randomized Controlled Trial
Source: Nutrients. 2026 Jun 28;18(13):2105. doi: 10.3390/nu18132105 (PMC13363418; doi:10.3390/nu18132105)
Supplement: Supplementary file 1 [file nutrients-18-02105-s001.zip › Supplementary Materials Figure S1.pdf]

Supplementary Materials

Figure S1. Forest Plot of Non-Inferiority Analyses on PG-SGA Score

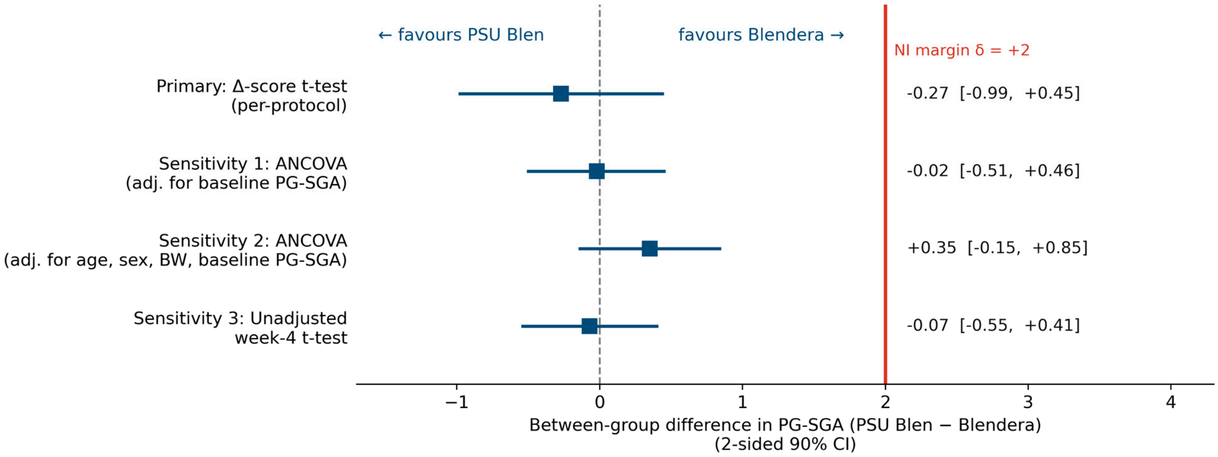

Figure S1. Forest plot of between-group differences (PSU Blen − Blendera) in the change in continuous PG-SGA score, displayed with two-sided 90% confidence intervals. Squares indicate point estimates; horizontal lines indicate two-sided 90% CIs. The solid red vertical line marks the prespecified non-inferiority margin (δ = +2.0 points); the dashed grey line marks the null value (no between-group difference). All four analyses (primary t-test on change scores; ANCOVA adjusted for baseline PG-SGA; ANCOVA adjusted for age, sex, body weight, and baseline PG-SGA; unadjusted week-4 t-test) yielded upper 90% CI bounds well below δ, supporting non-inferiority of PSU Blen.
